# Optimizing Open Radio Access Network Systems with LLAMA V2 for Enhanced Mobile Broadband, Ultra-Reliable Low-Latency Communications, and Massive Machine-Type Communications: A Framework for Efficient Network Slicing and Real-Time Resource Allocation

**DOI:** 10.3390/s24217009

**Published:** 2024-10-31

**Authors:** H. Ahmed Tahir, Walaa Alayed, Waqar ul Hassan, Thuan Dinh Do

**Affiliations:** 1 School of Computing, Engineering and Mathematics, Western Sydney University, Sydney 2751, Australia; 30067752@westernsydney.edu.au; 2 Department of Information Technology, College of Computer and Information Sciences, Princess Nourah Bint Abdulrahman University, Riyadh P.O. Box 84428, Saudi Arabia; 3 Department of Mathematics, Government College University, Lahore 54000, Pakistan; w.hassan@gcu.edu.pk; 4 School of Engineering, University of Mount Union, Alliance, OH 44601, USA; doth@mountunion.edu

**Keywords:** O-RAN, AI/ML, LLM, 5G, network slicing, resource allocation

## Abstract

This study presents an advanced framework integrating LLAMA_V2, a large language model, into Open Radio Access Network (O-RAN) systems. The focus is on efficient network slicing for various services. Sensors in IoT devices generate continuous data streams, enabling resource allocation through O-RAN’s dynamic slicing and LLAMA_V2’s optimization. LLAMA_V2 was selected for its superior ability to capture complex network dynamics, surpassing traditional AI/ML models. The proposed method combines sophisticated mathematical models with optimization and interfacing techniques to address challenges in resource allocation and slicing. LLAMA_V2 enhances decision making by offering explanations for policy decisions within the O-RAN framework and forecasting future network conditions using a lightweight LSTM model. It outperforms baseline models in key metrics such as latency reduction, throughput improvement, and packet loss mitigation, making it a significant solution for 5G network applications in advanced industries.

## 1. Introduction

**Figure 1:**
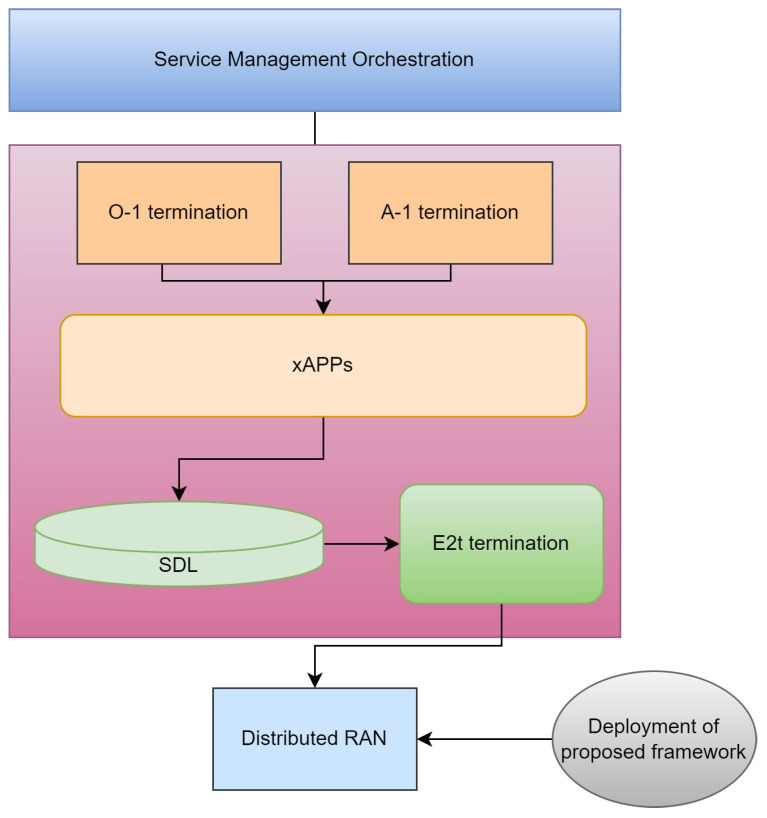
O-RAN big picture architecture and the point where the proposed framework will be deployed [2].

The technological evolution of wireless communication, mainly due to the availability of 5G and more, has resulted in an enhanced network architecture [1]. Another significant innovation is the Open Radio Access Network (O-RAN), a file, which—when virtualized and opened—becomes a system that offers better scalability and vendor dependencies [2]. O-RAN’s architecture separates hardware from software, allowing network operators to provide services such as eMBB, URLLC, and mMTC more efficiently [3]. Each of these services requires different levels of network performance, such as bandwidth, latency, and reliability, necessitating a highly adaptive and responsive management system. The architecture of O-RAN can be depicted in Figure 1.

Open Radio Access Network (O-RAN) represents a transformative shift in how modern wireless communication systems are designed and operated. Traditional Radio Access Network (RAN) architectures, such as those used in 4G and earlier generations, relied heavily on proprietary hardware and software from a limited number of vendors [4]. This led to an inflexible, highly integrated system that limited organizational adaptation and change. Nevertheless, O-RAN deviates from this approach by advocating openness, interoperability, and flexibility through its build-out and virtualization. It also gives the network operators the freedom to combine elements from different suppliers, creating a multi-vendor environment that is healthy for development and cost-effective.

Another crucial factor that should be mentioned while discussing O-RAN is the possibility of using network slicing [5], which will be essential for generation 5G and further. It allows for the construction of multiple virtualized segments of a network, or ‘slices’, which can be utilized for various services and applications. These slices can be fine-tuned to meet individual application requirements, such as eMBB, which requires high-speed broadband; URLLC, which requires deficient latency communication; or mMTC, which requires a high level of connectivity. This way, the operators can handle these slices dynamically, where RAN resources are granted according to the network conditions and given services. This flexibility is crucial for supporting emerging technologies like autonomous vehicles, smart cities, and the Internet of Things (IoT), which demand different levels of network performance [6].

The O-RAN architecture is based on principles of openness and standardization: it allows the integration of hardware and software sub-systems from different vendors. As a part of the O-RAN architecture, O-RAN Alliance, an industry group responsible for developing O-RAN standards, has identified critical elements like the RAN Intelligent Controller (RIC), that facilitates real-time decision making to optimize the network. The RIC is responsible for coordinating the operation of the network functions and uses machine learning and artificial intelligence techniques for efficient network resource provisioning, interference mitigation, and quality of service control across slices. The intelligent and data-oriented approach to managing networks distinguishes O-RAN from the more established RAN designs, enabling better adaptation to evolving and increasingly sophisticated wireless communications systems [2].

Hence, O-RAN has several benefits, but implementing the system has multiple issues. Fragmentation of network components and use of various vendors poses system integration and management challenges. In this case, integrating elements from different vendors is only possible due to immense standardization and subsequent testing. Further, the real-time decision making in O-RAN for various aspects—including establishing and managing network slices and different resources—further contributes to its complexity. Therefore, to counter these challenges, improved optimization methods like those presented in this paper are crucial for O-RAN systems to perform optimally. These techniques assist in controlling the complexity that arises from dynamic network slicing, cross-slice interference, and resource allocation to meet the various user needs within the network while achieving a high performance [7].

One of the critical problems in O-RAN is the control of the network slices—virtual partitions designed based on service requirements [8]. These slices need to coordinate in real time to optimize resource utilization, and the quality of the wireless network environment further exacerbates this issue, as the conditions are constantly in flux due to interference, variability in users’ traffic demands, or even hardware disparity. In response to this, the proposed method presents a mathematical approach comprising multiple layers, using stochastic partial differential equations (SPDEs) to capture the elements of the network. This approach considers other relations like interferences and cross-slice coupling, making it more comprehensive and realistic in predicting the network’s behavior.

In addition to the model’s comprehensive representation, a sophisticated control framework is introduced using coupled Hamilton–Jacobi–Bellman (HJB) equations. This framework manages decision making across the slices, dynamically adjusting the network policies based on real-time conditions. By embedding these policies into Riemannian manifolds, the system ensures smooth adjustments to network performance metrics, such as latency and reliability. The control framework also incorporates stability measurements to ensure that the system remains stable, even as conditions change, further improving network management under dynamic environments.

While large language models (LLMs) have been explored across various domains, their application within Open Radio Access Network (O-RAN) systems remains underdeveloped and limited in scope. This study presents a novel framework that integrates LLAMA_V2 into O-RAN, offering comprehensive applicability by enabling real-time network slicing, resource optimization, and explainable policy generation. Unlike previous methods that rely on traditional AI models or employ LLMs with restricted functionality, our framework leverages the unique strengths of LLAMA_V2 to address the complex challenges of dynamic resource management, cross-slice interference mitigation, and policy transparency in 5G networks. This approach demonstrates how tailored LLMs can significantly enhance the performance, adaptability, and scalability of multi-vendor O-RAN systems.

Furthermore, IoT devices mainly contribute through sensors that collect data. These devices are typically part of mMTC slices, which O-RAN supports to efficient communication in highly connected networks [9]. mMTC slices are critical for IoT applications, where a massive number of devices (like sensors) need to communicate with low-bandwidth, high-connectivity demands. O-RAN optimizes this with its dynamic network slicing, allowing the network to allocate appropriate resources depending on the service needs [10].

**Figure 2:**
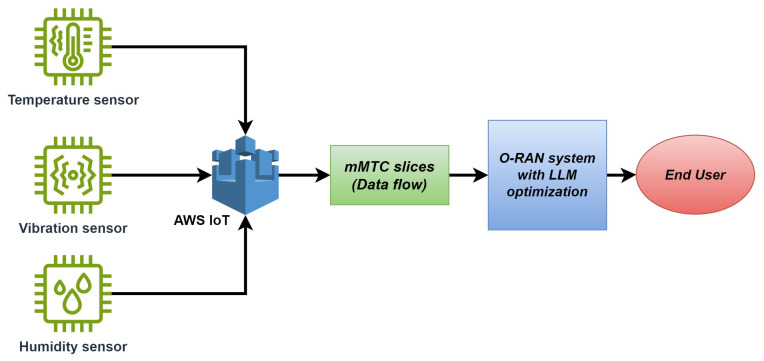
End-to-end proposed concept.

Sensors in IoT networks generate a constant flow of data, which must be efficiently managed. O-RAN’s with integrated large language models can dynamically allocate resources in an end-to-end framework from sensors to end users, as shown in Figure 2.

The proposed framework also has incorporated optimized and tiny machine learning to anticipate these network conditions so that the system can be prepared with the appropriate resources. Using the long short-term memory (LSTM) networks, future states are forecast according to historical data, and emergencies are corrected before they occur. This predictive ability is essential in wireless networks, since traffic and signal strength variations may significantly impact network performance. To address the interoperability issue when dealing with multiple vendors and slices, the model employs tensor decomposition methods to enhance the interaction process and integrate different parts seamlessly.

AI plays a crucial role in the Open Radio Access Network (O-RAN) by enabling the intelligent automation and optimization of network functions. It enhances dynamic network slicing, resource allocation, and traffic management, ensuring real-time adaptability to changing conditions. AI models, such as machine learning algorithms, are embedded in the RAN Intelligent Controller (RIC) to predict network behavior, reduce latency, and improve throughput. Additionally, AI-driven analytics provide insights for proactive fault detection, self-healing, and efficient energy management, making O-RAN a flexible and scalable solution for modern 5G networks.

LLAMA V2 excels in O-RAN systems by providing superior optimization, explainable policies, and scalability for real-time network slicing. In contrast, models like GPT-4 and GPT-NeoX offer high reasoning capabilities but lack the efficiency required for dynamic slicing. Traditional models such as DQN and PPO perform well in specific scenarios but struggle with scalability and adaptability across multiple slices. This comparison highlights LLAMA V2’s unique advantages, making it a more effective solution for O-RAN’s complex resource management challenges as shown in Table 1.

The evolution of communication technologies, particularly 5G and beyond, is critical for enabling advanced networks and applications. AI-driven technologies, fog computing, and certificate-less authentication schemes play pivotal roles in meeting the complex demands of modern communication networks.

AI-Driven Zero Touch Network and Service Management (ZSM) explores automation in 5G network management [11], focusing on the ETSI ZSM framework for closed-loop operations and reduced human intervention. The study emphasizes AI and machine learning (ML) for self-configuration, self-optimization, and fault tolerance in network operations. It highlights challenges such as the need for multi-domain management and the potential risks involved in fully automating network processes.

A CLA-FC5G authentication scheme paper introduces a certificate-less authentication mechanism that leverages fog computing in vehicular networks [12]. The framework aims to reduce communication overhead and improve security by enabling direct device-to-device (D2D) communication. The scheme provides strong resistance against known security threats like replay and impersonation attacks, ensuring real-time and scalable vehicular communication systems for 5G-enabled networks.

The PRIMATE framework proposes an AI-driven, context-aware profiling mechanism for resource management in 5G and beyond networks. It utilizes big data classification to predict the behavior of devices and users for proactive network management. The study integrates hierarchical clustering and supervised learning techniques to generate behavioral profiles, supporting applications in Industry 4.0 with enhanced resource planning and reduced latency [13].

The main weaknesses across these studies include the ZSM framework’s complexity and the risks associated with over-automation, the CLA-FC5G scheme’s potential vulnerability to emerging security threats, and PRIMATE’s dependence on large datasets, which could reduce its effectiveness in data-scarce environments.

The framework has been designed to handle real-world challenges with resilience. It leverages probabilistic techniques to assess performance in uncertain conditions, ensuring the network remains reliable within acceptable limits. Sensitivity analysis helps fine-tune the model’s parameters, allowing it to perform optimally across different scenarios. By blending advanced mathematical models, real-time control systems, machine learning, and robustness analysis, this approach provides a well-rounded solution for managing O-RAN systems. Ultimately, it is built to efficiently meet the evolving demands of modern wireless networks.

## 2. Motivation and Contributions

The development of 5G and O-RAN networks provides users with unprecedented freedom and flexibility, enabling a wide range of applications such as eMBB, URLLC, and mMTC. However, the dynamic management of these services, while ensuring quality and reliability, remains a challenge due to real-time resource management, diverse vendors, and network slicing. The traditional RAN structure, which involves closed and proprietary equipment and software, limits flexibility and increases operational costs. In contrast, O-RAN promotes openness and offers an architecture where hardware can be selected from individual suppliers. However, O-RAN introduces new technical challenges, such as determining how to mitigate cross-slice interference, implement dynamic policies, and efficiently allocate resources.

The motivation for this research arises from addressing these technical challenges in O-RAN. A mathematical model based on stochastic partial differential equations (SPDEs) is proposed to efficiently capture network dynamics and account for inter-layer interactions within network conditions. A Hamilton–Jacobi–Bellman (HJB) control framework is employed to make optimal decisions regarding slices, while long short-term memory (LSTM) networks are used for network forecasting. Additionally, the nine uncertainties are addressed, and the real-world characteristics of the model are demonstrated through advanced tensor decomposition and sensitivity analysis.

This study is introduces a comprehensive and novel approach for managing the dynamic and multi-vendor environment of O-RAN systems. By combining advanced control frameworks with predictive modeling and optimization techniques, this research tackles the core challenges of interoperability, performance stability, and resource allocation—critical for the successful deployment and management of next-generation wireless networks. Following are the three key contributions of this study:

- To develop a novel multi-layered mathematical model for O-RAN systems, incorporating network slicing with distinct service requirements (eMBB, URLLC, mMTC). This model leverages stochastic partial differential equations (SPDEs) to dynamically represent the state evolution of network elements within each slice, along with inter-slice coupling and cross-slice interference quantification, enabling a detailed and realistic representation of modern wireless networks.
- To introduce a sophisticated control framework utilizing coupled Hamilton–Jacobi–Bellman (HJB) equations for optimal decision making across network slices. The framework integrates policy embeddings derived from LLAMA V2 into Riemannian manifolds, applying geodesic distances and curvature tensors to rigorously enforce and adapt network policies. Additionally, constrained optimization techniques using Karush–Kuhn–Tucker (KKT) conditions ensure adherence to operational limits and quality of service (QoS) requirements, while dynamic resource allocation is managed through adaptive control laws based on Lyapunov stability theory.
- To establish a multi-objective optimization framework to analyze and optimize interoperability across network slices and vendors, utilizing CANDECOMP/PARAFAC tensor decomposition. The framework includes machine-learning-based prediction using LSTM networks for forecasting network conditions, enabling proactive policy adjustments. Rigorous robustness analysis and sensitivity quantification were performed to ensure system resilience under uncertainty, with theoretical performance bounds providing a strong foundation for real-world implementation and benchmarking.

## 3. Literature Review

The use of wireless signals for communications has changed and improved from a simple and closed base structure to a more complex and open one. Among the more notable advancements in this area, the O-RAN system, which has rapidly become crucial to advancing next-generation wireless networks, including 5G and beyond, can be singled out. O-RAN promises to integrate an open, premises-based, virtualized system that facilitates flexibility, integration, and scalability among multiple players from different vendors. O-RAN’s separation of hardware and software components allows network operators to optimize functions based on their needs, which benefits from a multi-vendor environment [14].

The significance of O-RAN lies in its potential to enhance network performance while supporting diverse use cases such as enhanced Mobile Broadband (eMBB), Ultra-Reliable Low-Latency Communications (URLLC), and massive Machine-Type Communications (mMTC). These use cases demand varying levels of Quality of Service (QoS), which necessitates a dynamic and adaptive network architecture. O-RAN’s openness and programmability allow for real-time adjustments to network resources, making it a key enabler for advanced applications like autonomous vehicles, smart cities, and the Internet of Things (IoT) [15].

Some of the challenges evident in the implementation of O-RAN include network slicing, which is reported as one of the most significant issues [14]. In an O-RAN setting, network slicing refers to the creation of multiple logical network partitions, or slices, for differentiated services. These slices must be dynamically controlled and adapted in terms of resource allocation to ensure Quality of Service (QoS) and enable interoperability in a multi-vendor environment. Achieving this is challenging due to cross-slice interference, response time constraints, and dynamic topological changes.

Another significant challenge is the need for real-time decision-making and control [16]. O-RAN systems must be capable of making split-second decisions on resource allocation, network optimization, and fault management to ensure seamless service delivery. The decentralized and disaggregated nature of O-RAN increases the difficulty of achieving this, as network functions are distributed across various components and vendors. Moreover, the inherent uncertainty in wireless environments, such as varying signal strengths and unpredictable user behavior, adds to the complexity of managing O-RAN systems effectively.

Interoperability across multiple vendors is another critical issue; while O-RAN promotes a multi-vendor ecosystem [17], ensuring that different components from various vendors work together seamlessly is a significant challenge. This requires sophisticated mechanisms for compatibility, performance optimization, and troubleshooting. Additionally, the need for robust security measures and the protection of sensitive data in a highly interconnected and open environment cannot be overstated.

Given the complexity of O-RAN systems, especially in the context of network slicing and real-time control, large language models (LLMs) like LLAMA V2 present a promising solution. LLAMA V2, with its advanced capabilities in natural language processing and understanding, offers several advantages that make it particularly well-suited for this domain. Comprehensive comparison of LLAMA V2 with other state of the art models can be depicted in Table 2 [18,19,20,21,22,23,24,25,26,27,28,29,30,31,32,33,34,35,36], with trade off reasons to choose LLAMA V2 for network slicing among other language models.

LLAMA V2 can process and analyze vast amounts of data from different network slices, making it possible to derive optimal decisions in real time. By integrating LLAMA V2 into O-RAN, network operators can leverage its ability to understand and synthesize information across multiple sources, enabling more informed and accurate control decisions. The model’s capacity to solve coupled Hamilton–Jacobi–Bellman (HJB) equations ensures that optimal control policies are derived for each network slice, minimizing latency and maximizing resource utilization.

One of the standout features of LLAMA V2 is its ability to generate explanations and outputs in natural language. This capability is crucial in an O-RAN environment, where network decisions need to be transparent and understandable by operators and stakeholders. LLAMA V2 can translate complex technical metrics and optimization results into human-readable reports, providing clear insights into network performance and the rationale behind specific decisions. This not only enhances operational efficiency but also facilitates troubleshooting and continuous improvement.

LLAMA V2’s training framework, which incorporates policy embeddings into high-dimensional Riemannian manifolds, allows it to adapt policies dynamically across different network slices. This adaptability is essential for managing the diverse requirements of eMBB, URLLC, and mMTC services in a unified manner. Additionally, LLAMA V2’s ability to handle tensor decomposition and multi-objective optimization ensures that it can manage the interoperability challenges inherent in a multi-vendor O-RAN system. By optimizing the Pareto front across slices and vendors, LLAMA V2 ensures that the network operates at peak efficiency while maintaining the necessary balance between competing performance metrics.

LLAMA V2’s integration with machine-learning-based prediction models, such as LSTM networks, enhances its ability to forecast future network conditions and adjust control policies proactively. This predictive capability is particularly valuable in dynamic and unpredictable wireless environments, where anticipatory actions can prevent performance degradation and ensure consistent QoS. By continuously learning from historical data and adapting to real-time inputs, LLAMA V2 enables O-RAN systems to remain resilient and responsive under varying conditions.

In conclusion, O-RAN systems represent a significant advancement in wireless communications, offering the flexibility and scalability needed to support diverse and demanding use cases. However, the complexity of managing network slices, ensuring real-time decision-making, and maintaining interoperability across vendors presents substantial challenges. LLAMA V2, with its advanced decision-making capabilities, adaptability, and ability to provide natural language explanations, emerges as a powerful tool to address these challenges. By leveraging LLAMA V2, O-RAN systems can achieve optimal performance, enhanced transparency, and robust adaptability, paving the way for the next generation of wireless networks, as shown in Table 3 [37,38,39,40,41,42,43,44,45,46,47,48,49].

## 4. Proposed Methodology

**Figure 3:**
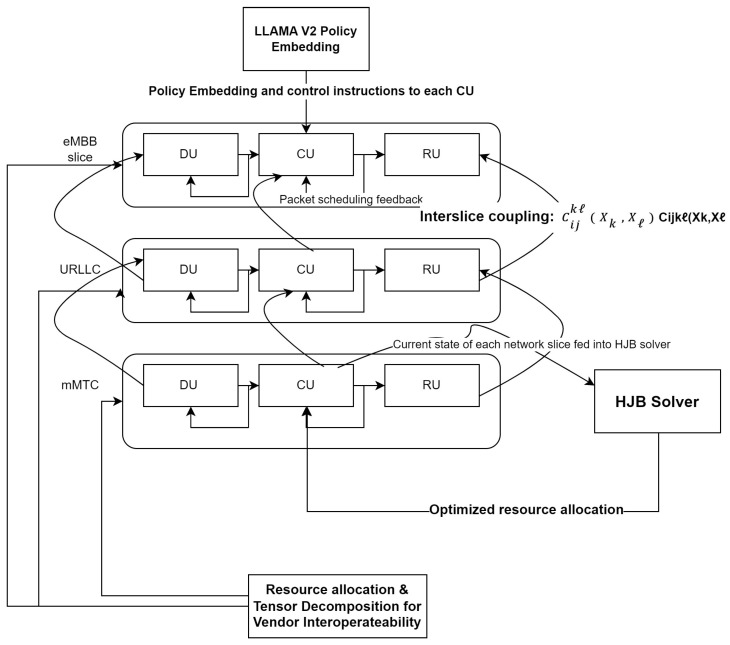
Proposed methodology.

The proposed methodology depicted in Figure 3 for integrating large language models (LLMs), specifically LLAMA V2, into O-RAN systems and wireless communications with a focus on network slicing employs advanced mathematical models, optimization techniques, and rigorous technical approaches to create a highly adaptive, scalable, and efficient framework.

We begin by representing the O-RAN architecture using a multi-layer, directed, weighted multigraph $G={\left\{G_{k}\right\}}_{k=1}^{K}$, where each layer $G_{k}=\left(V_{k},E_{k},W_{k}\right)$ corresponds to a distinct network slice *k* (e.g., eMBB, URLLC, mMTC). Each vertex $v_{i}^{k}\in V_{k}$ represents a network element (DU, CU, RU), and each directed edge $e_{ij}^{k}\in E_{k}$ represents a communication link between these elements, with weights $w_{ij}^{k}$ indicating link capacities or delays.

The state of each network element in slice *k* evolves according to a system of coupled stochastic partial differential equations (SPDEs):

(1) $$\begin{array}{cc} \frac{\partial X_{i}^{k}\left(t,x\right)}{\partial t}+L_{k}X_{i}^{k}\left(t,x\right)= & \;\mu_{i}^{k}\left(t,X_{i}^{k}\left(t,x\right),X_{-i}^{k}\left(t,x\right)\right)\\ \; & +\sum_{j=1}^{m}\sigma_{ij}^{k}\left(t,X_{i}^{k}\left(t,x\right),X_{j}^{k}\left(t,x\right)\right)\frac{\partial W_{j}\left(x\right)}{\partial t}\\ \; & +\nu_{ij}^{k}\left(t,X_{i}^{k}\left(t,x\right)\right) \end{array}$$

- $X_{i}^{k}\left(t,x\right)$: The state variable representing the value of a network element *i* at time *t* and location *x* within a slice *k*.
- $\mu_{i}^{k}$: Drift term describing the deterministic evolution of the network state, capturing how the state evolves under normal conditions.
- $\sigma_{ij}^{k}$: Volatility term that models stochastic fluctuations in the state due to unpredictable factors (e.g., signal interference).
- $W_{j}\left(x\right)$: A Wiener process representing a random noise source, often used in stochastic models to capture uncertainty.
- $\nu_{ij}^{k}$: A perturbation term accounting for disturbances or random effects in the network.

where $L_{k}$ is a differential operator representing the interactions within slice *k*, $W_{j}\left(x\right)$ are independent spatial Wiener processes, and $\nu_{ij}^{k}$ is a noise term representing random perturbations.

The interactions between slices are captured by an inter-slice coupling term:

(2) $$C_{ij}^{k\ell }\left(X_{k},X_{\ell }\right)=\sum_{\ell \neq k}\int_{\Omega }\gamma_{ij}^{k\ell }\left(r\right)X_{i}^{k}\left(t,r\right)X_{j}^{\ell }\left(t,r\right)\;dr$$

- $E_{u^{k}}$: Expectation operator, representing the expected value over the control inputs $u^{k}$.
- $u_{kk}$: Control policy parameters specific to slice *k*, used to determine resource allocations.
- ${\mathrm{Cost}}_{i}^{k}\left(u\right)$: The cost function associated with control decisions for a given slice *k*, which may include metrics like latency, energy consumption, and resource utilization.
- λ and α: Regularization parameters that balance the different components of the loss function, ensuring generalization and stability during training.

where $\gamma_{ij}^{k\ell }\left(r\right)$ is a kernel function representing the coupling strength between slices *k* and *ℓ*, and Ω is the spatial domain of the network.

The policy embeddings derived from LLAMA V2 are mapped into a Riemannian manifold $M_{k}$ specific to each slice *k*. The manifold is equipped with a Riemannian metric tensor $g_{ij}^{k}\left(v\right)$, and the geodesic distance between two embeddings $v_{1}^{k}$ and $v_{2}^{k}$ is defined by:

(3) $$d\left(v_{1}^{k},v_{2}^{k}\right)=\underset{\gamma_{k}}{\inf}\int_{0}^{1}g_{ij}^{k}\left(\gamma_{k}\left(t\right)\right)\frac{\partial \gamma_{k}^{i}}{\partial t}\frac{\partial \gamma_{k}^{j}}{\partial t}\;dt$$

where $\gamma_{k}\left(t\right)$ is a geodesic curve on $M_{k}$. The curvature of the manifold, given by the Riemann curvature tensor $R_{ijkl}^{k}$, affects the sensitivity of policy enforcement:

(4) $$R_{ijkl}^{k}=\frac{\partial g_{il}^{k}}{\partial v_{j}}-\frac{\partial g_{jl}^{k}}{\partial v_{i}}+g_{mn}\left(\Gamma_{im}^{k}\Gamma_{jn}^{k}-\Gamma_{jm}^{k}\Gamma_{in}^{k}\right)$$

where $\Gamma_{ij}^{k}$ are the Christoffel symbols associated with $g_{ij}^{k}$.

The training of LLAMA V2 is driven by a loss function that incorporates both data-driven and regularization components:

(5) $$\begin{array}{cc} L(\theta )= & -\sum_{k=1}^{K}\sum_{t=1}^{T}\log p\left(y_{t}^{k}|y_{1:t-1}^{k},x_{k};\theta \right)\\ \; & +\lambda \sum_{k=1}^{K}E_{u_{k}}\left[\sum_{i=1}^{N}{\mathrm{Cost}}_{i}^{k}\left(u_{k}\right)+\frac{1}{2}\sum_{k=1}^{K}{∥u_{kk}∥}^{2}\right]\\ \; & +\alpha \sum_{k=1}^{K}∥\nabla_{\theta }L_{\mathrm{baseline}}^{k}\left(\theta \right)∥ \end{array}$$

where $L_{\mathrm{baseline}}^{k}\left(\theta \right)$ represents the loss from a baseline model used to guide LLAMA V2’s training.

The control inputs $u_{k}\left(t\right)$ for each slice *k* are determined by solving a coupled system of Hamilton–Jacobi–Bellman (HJB) equations:

(6) $$\frac{\partial V_{k}\left(X_{k},t\right)}{\partial t}+\min_{u_{k}\left(t\right)}\left[H_{k}\left(X_{k}\left(t\right),u_{k}\left(t\right),\nabla_{X_{k}}V_{k}\left(X_{k},t\right)\right)\right]=0,\;k=1,2,\cdots ,K$$

where $V_{k}\left(X_{k},t\right)$ is the value function for slice *k*, representing the expected cost-to-go from state $X_{k}\left(t\right)$, and $H_{k}$ is the Hamiltonian for slice *k*, which is given by:

(7) $$H_{k}\left(X_{k},u_{k},\nabla_{X_{k}}V_{k}\right)=\sum_{i=1}^{N_{k}}\left(\mu_{i}^{k}\left(X_{k}\right)\nabla_{X_{i}^{k}}V_{k}+\frac{1}{2}\mathrm{Tr}\left(\sigma_{i}^{k}\left(X_{k}\right)\sigma_{i}^{k⊤}\left(X_{k}\right)\nabla_{X_{i}^{k}}^{2}V_{k}\right)\right)+{\mathrm{Cost}}_{i}^{k}\left(u_{k}\right)$$

The HJB equations are coupled through the inter-slice coupling terms $C_{ij}^{k\ell }\left(X_{k},X_{\ell }\right)$ from Equation (2), resulting in a complex, high-dimensional optimization problem. The solution to the HJB equations provides the optimal control policy $u_{k}\left(t\right)$ for each slice.

The optimization problem for determining the control inputs $u_{k}\left(t\right)$ is subject to a set of inequality constraints representing operational limits and quality of service (QoS) requirements for each slice:

(8) $$g_{j}^{k}\left(u_{k}\right)\leq 0,\;j=1,2,\cdots ,M_{k}$$

These constraints are enforced using the Karush–Kuhn–Tucker (KKT) conditions, where the objective function is given by:

(9) $$\min_{u_{k},\lambda_{k}}\left[\sum_{k=1}^{K}\sum_{i=1}^{N}\left(\alpha_{i}^{k}\cdot {\mathrm{Latency}}_{i}^{k}\left(u_{k}\right)+\beta_{i}^{k}\cdot {\mathrm{ErrorRate}}_{i}^{k}\left(u_{k}\right)\right)+\sum_{j=1}^{M_{k}}\lambda_{j}^{k}\cdot g_{j}^{k}\left(u_{k}\right)\right]$$

The Lagrange multipliers $\lambda_{j}^{k}$ are determined by solving the KKT system:

(10) $$\nabla_{u_{k}}L_{k}\left(u_{k},\lambda_{k}\right)=0,\;\lambda_{j}^{k}g_{j}^{k}\left(u_{k}\right)=0,\;\lambda_{j}^{k}\geq 0,\;g_{j}^{k}\left(u_{k}\right)\leq 0$$

where $L_{k}\left(u_{k},\lambda_{k}\right)$ is the Lagrangian for slice *k*.

Interoperability across multiple vendors and network slices is achieved through multi-objective optimization. The Pareto front $P_{k}$ for each slice *k* is identified by solving:

(11) $$P_{k}=\left\{u_{k}\in U_{k}|∄u_{k}^{\prime }\in U_{k}:F_{k}\left(u_{k}^{\prime }\right)\leq F_{k}\left(u_{k}\right),F_{k}\left(u_{k}^{\prime }\right)\neq F_{k}\left(u_{k}\right)\right\},\;k=1,2,\cdots ,K$$

where $F_{k}\left(u_{k}\right)$ represents the vector of objective functions for slice *k*, including metrics such as latency, error rate, and resource utilization.

To analyze interoperability across slices and vendors, tensor decomposition techniques are applied. The performance metrics across all slices are represented by a tensor $T\in R^{K\times M\times N\times P}$, where *K* is the number of slices, *M* is the number of vendors, *N* is the number of performance metrics, and *P* is the number of test cases. The tensor is decomposed using CANDECOMP/PARAFAC (CP) decomposition:

(12) $$T\approx \sum_{r=1}^{R}a_{r}^{k}\circ b_{r}^{m}\circ c_{r}^{n}\circ d_{r}^{p}$$

where ∘ denotes the outer product, and $a_{r}^{k},b_{r}^{m},c_{r}^{n},d_{r}^{p}$ are the factors corresponding to slices, vendors, performance metrics, and test cases, respectively.

The evaluation and benchmarking of the proposed framework involve deriving rigorous theoretical performance bounds using advanced probabilistic techniques. For example, Chernoff bounds are used to derive tail probabilities for latency and error rates across slices:

(13) $$P\left(X_{k}\geq \mu_{k}+\delta_{k}\right)\leq \exp\left(-\frac{{\left(\delta_{k}\right)}^{2}}{2(\sigma_{k}^{2}+\delta_{k}/3)}\right),\;k=1,2,\cdots ,K$$

where $\mu_{k}$ is the mean latency, $\sigma_{k}^{2}$ is the variance, and $\delta_{k}$ represents the deviation from the mean for slice *k*.

Hypothesis testing using the likelihood ratio test is employed to compare the LLAMA V2-driven control system with baseline methods:

(14) $$\Lambda_{k}=2\left[\sum_{k=1}^{K}\left(\log L_{k}\left({\hat{\theta }}^{1}\right)-\log L_{k}\left({\hat{\theta }}^{0}\right)\right)\right]$$

where $L_{k}\left({\hat{\theta }}^{1}\right)$ and $L_{k}\left({\hat{\theta }}^{0}\right)$ represent the maximum likelihood estimates under the alternative and null hypotheses for slice *k*.

Dynamic resource allocation across network slices is managed through an adaptive control framework, where resources such as bandwidth, power, and computational capacity are dynamically allocated based on the real-time requirements of each slice. The resource allocation problem is formulated as a constrained optimization problem:

(15) $$\max_{r_{k}\left(t\right)}\sum_{k=1}^{K}U_{k}\left(r_{k}\left(t\right)\right)\;\mathrm{subject}\;\mathrm{to}\;\sum_{k=1}^{K}r_{k}\left(t\right)\leq R\left(t\right),\;r_{k}\left(t\right)\geq 0$$

where $r_{k}\left(t\right)$ is the resource vector for slice *k* at time *t*, $U_{k}\left(r_{k}\left(t\right)\right)$ is the utility function representing the QoS for slice *k*, and R(t) is the total available resource vector.

The adaptive control law is derived using Lyapunov stability theory, ensuring that the system remains stable under dynamic conditions. The Lyapunov candidate function V(r(t)) is defined as:

(16) $$V\left(r\left(t\right)\right)=\frac{1}{2}\sum_{k=1}^{K}{∥r_{k}\left(t\right)-r_{k}^{\mathrm{opt}}\left(t\right)∥}^{2}$$

where $r_{k}^{\mathrm{opt}}\left(t\right)$ is the optimal resource allocation for slice *k*. The control law is then given by:

(17) $$\frac{dr_{k}\left(t\right)}{dt}=-\nabla_{r_{k}}V\left(r\left(t\right)\right)+g_{k}\left(t\right)$$

where $g_{k}\left(t\right)$ is a feedback term that adjusts the resource allocation based on real-time network conditions.

The robustness of the proposed framework is analyzed by quantifying the impact of uncertainties in network parameters and environmental conditions. Uncertainties in the system are modeled using a stochastic perturbation term ξ(t), leading to the following perturbed SPDE for the state evolution:

(18) $$\frac{\partial X_{i}^{k}\left(t,x\right)}{\partial t}+L_{k}X_{i}^{k}\left(t,x\right)=\mu_{i}^{k}\left(t,X_{i}^{k}\left(t,x\right),X_{-i}^{k}\left(t,x\right)\right)+\sum_{j=1}^{m}\sigma_{ij}^{k}\left(t,X_{i}^{k}\left(t,x\right),X_{j}^{k}\left(t,x\right)\right)\frac{\partial W_{j}\left(x\right)}{\partial t}+\xi_{i}^{k}\left(t,x\right)$$

The impact of these uncertainties on the system’s performance is quantified using sensitivity analysis, where the sensitivity of the performance metrics $F_{k}\left(u_{k}\right)$ to the uncertainties is given by:

(19) $$S_{ij}^{k}=\frac{\partial F_{i}^{k}\left(u_{k}\right)}{\partial \xi_{j}^{k}},\;i=1,2,\cdots ,N,\;j=1,2,\cdots ,P$$

where $S_{ij}^{k}$ represents the sensitivity of the *i*-th performance metric in slice *k* to the *j*-th uncertainty.

Tiny and optimized ML models are employed to predict and forecast future network conditions and performance metrics, enabling dynamic adjustments to control policies. The prediction model is fine-tuned on historical data using LSTM:

(20) $${\hat{X}}_{k}\left(t+1\right)=\mathrm{LSTM}\left(X_{k}\left(t\right),h_{k}\left(t\right)\right)$$

where ${\hat{X}}_{k}\left(t+1\right)$ is the predicted state for slice *k* at time t+1, and $h_{k}\left(t\right)$ is the hidden state of the LSTM network.

Predicted states are used to update control policies as follows:

(21) $$u_{k}\left(t+1\right)=u_{k}^{\mathrm{opt}}\left({\hat{X}}_{k}\left(t+1\right)\right)$$

The proposed mathematical framework integrates advanced concepts to improve model performance and logical interfacing between O-RAN components. Using this multi-layered model, resource allocation and network slicing in dynamic environments can bring significant enhancement, ensuring reliable communication.

Table 4 provides definitions and descriptions of key symbols and variables used in the mathematical formulations throughout the paper. This ensures consistency and facilitates precise interpretation of the equations:

## 5. Experimental Setup

The experimental setup starts with NS-3, an open-source network simulation tool for modeling the O-RAN architecture. NS-3 emulates network devices like distributed, central, and radio units. It coordinates these elements and produces traffic information and fundamental system values, including throughput, delay, and resource availability. The result obtained from NS-3 is exported to MATLAB for further signal processing and analysis. Another reason is the ability of MATLAB PDE Toolbox to use stochastic partial differential equations (SPDEs) to model the dynamic progression of the state of individual elements in the network to emulate inter-slice interference and link quality. Interactions between NS-3 and MATLAB are based on data interchange methods that enable simulation and real-time state representation. The next step is to apply the concepts in the LLAMA V2 model for the efficient use of resources and using fine-tuning in PyTorch. Due to the high complexity and size of data used for O-RAN simulations, the model is trained on NVIDIA Tesla V100 GPUs. With 32 GB memory and a maximum of 125 teraFLOPS, these GPUs speed up the training process because large-scale neural networks require parallel processing. Once trained, the LLAMA V2 model is saved from PyTorch in pt format and then transferred to MATLAB simulation, which gives real-time decisions about allocating resources in different network slices.

Further, TensorFlow is used to run LSTM networks, through which the future state of the network, for instance, traffic, and latency, is anticipated. Such predictions enable the system to modify the resource allocation plan before it is implemented. The LSTM models are trained with traffic data produced using NS-3 simulations and improve the system’s capacity to make anticipative modifications.

For constrained optimization problems, the Gurobi Optimizer is incorporated within the experimental framework. Thus, Gurobi solves the Karush–Kuhn–Tucker (KKT) optimization problem, provided that resource constraints and quality of service requirements are satisfied for each network slice. MATLAB interacts with Gurobi in the background directly through the optimization toolbox of MATLAB; Gurobi then solves the optimal resource allocation strategies using the input data from the SPDEs and the predictive LSTM models. The data generated from all system components, such as NS-3, MATLAB, PyTorch, and TensorFlow, is processed and analyzed using Pandas and visualized through Matplotlib. Pandas is used to handle and clean the large datasets generated from real-time simulations, while Matplotlib is employed to create graphs illustrating performance metrics such as throughput, latency, and packet loss. This allows a clear visualization of the system’s performance under various conditions.

The entire experimental pipeline, from NS-3 simulations, mathematical modeling in MATLAB, machine learning in PyTorchand TensorFlow, optimization with Gurobi, and data visualization with Pandas and Matplotlib, ensures a comprehensive testing environment.

## 6. Results

The baseline model used for comparison is a conventional Deep Q-Network (DQN), commonly applied in resource allocation and network slicing; while the DQN is effective in isolated or static scenarios, it lacks the capability to handle the dynamic, multi-slice dependencies, and cross-slice interferences present in O-RAN systems. Additionally, the baseline model does not offer explainable outputs, limiting its transparency and adaptability in real-time environments. In contrast, our proposed framework integrates LLAMA_V2, LSTM-based forecasting, and HJB equations, providing a comprehensive solution to these challenges.

Key metrics were employed to evaluate the performance of the proposed model. Decision accuracy measures the percentage of correct control decisions compared to known optimal solutions under varying network conditions. Policy explainability is quantified using LLAMA_V2’s natural language outputs, which were assessed through human feedback and SHAP-based metrics to gauge transparency. Prediction accuracy evaluates how closely the LSTM forecasts align with actual network states using mean squared error (MSE), while error rate represents the proportion of incorrect decisions relative to the total.

Although the observed improvements in packet loss ranged between 0.5 and 3.0%, even such marginal gains are significant in high-traffic O-RAN systems. A small reduction in packet loss can lead to fewer retransmissions and better quality of service (QoS), providing substantial benefits at scale.

While the baseline model could be further trained or fine-tuned, it faces architectural limitations that prevent it from matching the performance of the proposed framework. The baseline DQN model lacks the predictive power offered by LSTM forecasting and the advanced control mechanisms enabled by HJB equations. Furthermore, the policy explainability provided by LLAMA_V2 cannot be achieved without major modifications to the baseline. The proposed framework delivers not only superior performance across multiple metrics but also the transparency needed for effective real-time decision making, making it a robust and innovative solution for O-RAN systems.

**Figure 4:**
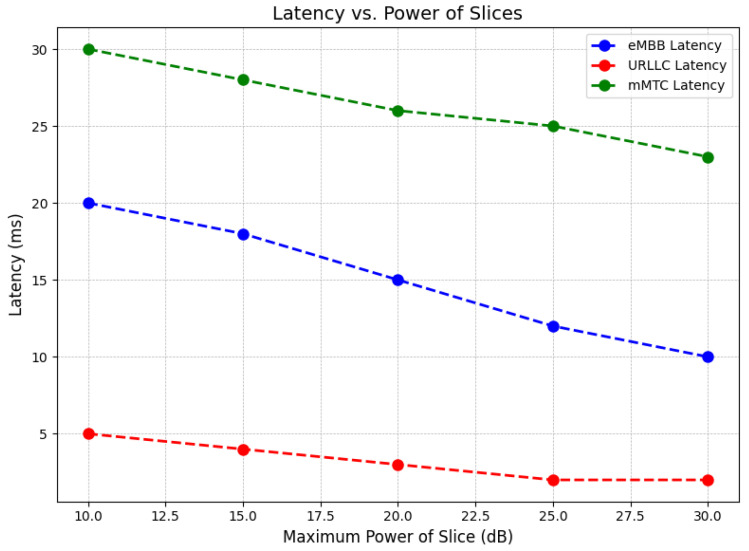
Latency reduction as a function of increasing power for eMBB, URLLC, and mMTC services in 5G networks.

The graph in Figure 4 shows the relationship between latency and the power of 5G network slices for eMBB, URLLC, and mMTC services. As the power increases, latency decreases for all three services. Specifically, eMBB latency decreases from 20 ms to 10 ms, URLLC starts at around 5 ms and drops to 2 ms, and mMTC latency decreases from 30 ms to 20 ms. URLLC consistently has the lowest latency, making it suitable for real-time applications. eMBB has moderate latency, while mMTC has the highest latency, which is fitting for IoT applications. This indicates that power management plays a crucial role in optimizing latency, with higher power improving latency performance across all services.

**Figure 5:**
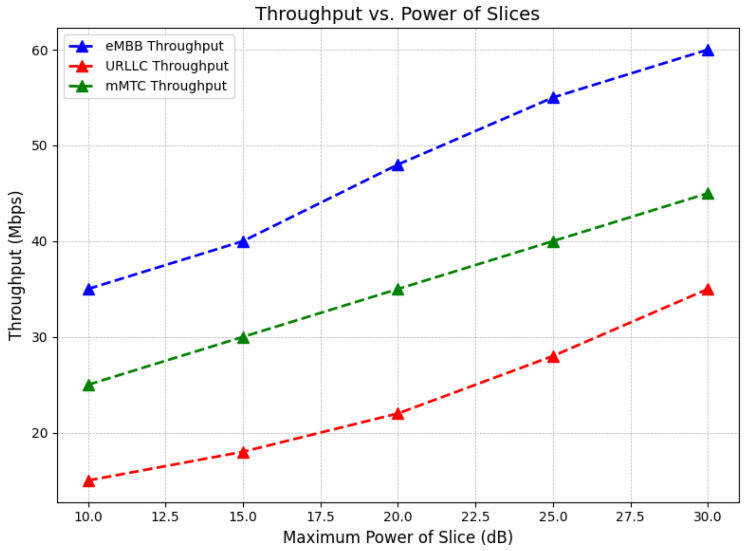
Throughput is increased with rising power for eMBB, URLLC, and mMTC services in 5G networks.

The changes in throughput as power allocated to 5G network slices increases are illustrated in Figure 5. Three types of services are compared: eMBB (enhanced mobile broadband), URLLC (ultra-reliable low-latency communication), and mMTC (massive machine-type communication). The maximum power of each slice is shown on the x-axis in decibels (dB), ranging from 10 dB to 30 dB, while throughput in megabits per second (Mbps) is displayed on the y-axis. Throughput is observed to increase consistently across all services as power is raised, with eMBB achieving the highest throughput, followed by mMTC and URLLC.

**Figure 6:**
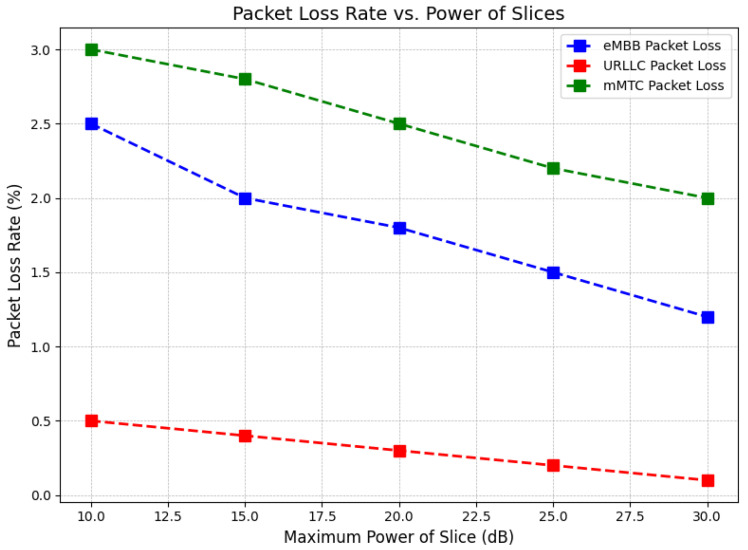
Packet loss decreases with increasing power for eMBB, URLLC, and mMTC services in 5G networks.

The graph in Figure 6 shows how packet loss rate varies as the power allocated to 5G network slices increases for eMBB, URLLC, and mMTC services. The x-axis represents the maximum power of each slice in decibels (dB), ranging from 10 dB to 30 dB, while the y-axis shows packet loss rate as a percentage. The three services—eMBB (enhanced mobile broadband), URLLC (ultra-reliable low-latency communication), and mMTC (massive machine-type communication)—are represented with distinct dashed lines. As the power of each slice increases, packet loss decreases for all three services. eMBB packet loss starts at approximately 2.0% and gradually drops to around 0.8%. URLLC experiences the lowest packet loss, starting near 0.5% and approaching 0% as power increases. In contrast, mMTC shows the highest packet loss, beginning at about 3.0% and reducing to just above 2.0%. This shows that higher power allocation reduces packet loss rates across all services. URLLC benefits the most from increased power, maintaining the lowest packet loss rates, followed by eMBB and mMTC. This suggests that URLLC is the most reliable in terms of data transmission, while mMTC experiences the most packet loss, possibly due to the nature of massive machine-type communications, which involves a large number of devices. Proper power management can therefore significantly improve packet transmission reliability, especially in services requiring low-latency and high reliability like URLLC.

**Figure 7:**
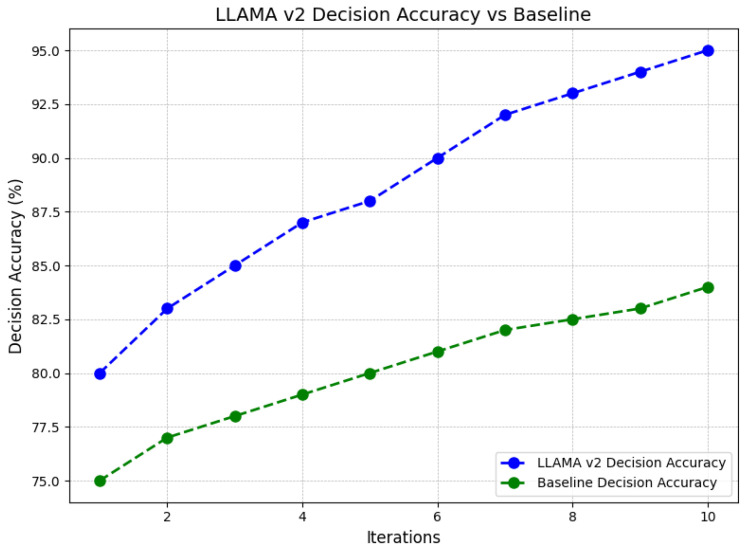
Comparison of decision accuracy between LLAMA V2 and baseline over iterations.

Figure 7 compares the decision accuracy of LLAMA V2 against a baseline model over 10 iterations. The x-axis represents the number of iterations, and the y-axis shows decision accuracy in percentage. It is clear that LLAMA V2 consistently performs better than the baseline model across all iterations. Both models demonstrate improvement in accuracy as the number of iterations increases, but LLAMA V2 exhibits a faster and more significant rise. At the beginning, LLAMA V2 starts with a decision accuracy of around 80%, quickly improving to approximately 95% by the tenth iteration. In contrast, the baseline model starts at 75% accuracy and sees a more gradual improvement, reaching only about 82.5% by the tenth iteration. The widening gap between the two models suggests that LLAMA V2 not only provides better initial accuracy but also adapts more effectively with additional iterations, leading to a more reliable decision-making process. This analysis highlights the superior performance of LLAMA V2 over the baseline, with its accuracy improving more consistently and at a higher rate, making it a more effective choice for tasks requiring high decision accuracy.

**Figure 8:**
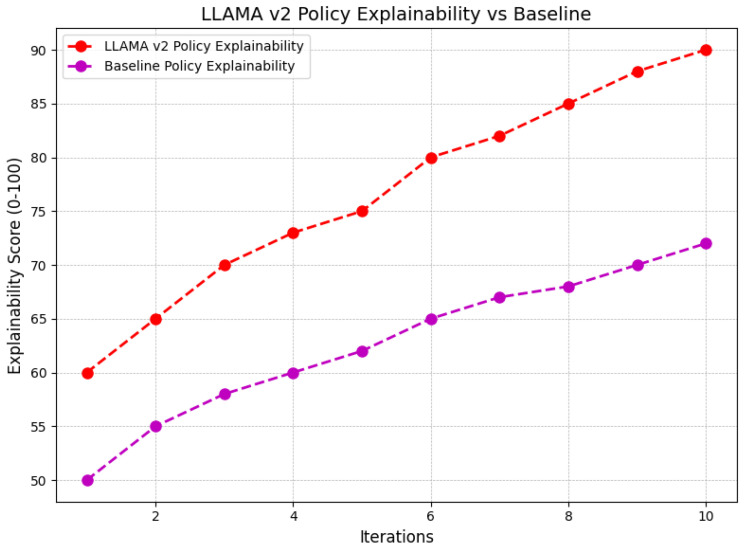
Comparison of policy explainability scores between LLAMA V2 and baseline over iterations.

The explainability score of policies generated by LLAMA V2 is compared with a baseline model over 10 iterations in the graph. The number of iterations is represented on the x-axis, while the explainability score, on a scale from 0 to 100, is displayed on the y-axis as shown in Figure 8. LLAMA V2 demonstrates consistently higher explainability scores than the baseline across all iterations, with both models improving as iterations progress. LLAMA V2 begins with an explainability score of approximately 60, steadily increasing to nearly 90 by the tenth iteration. In contrast, the baseline model starts with a score of around 50 and gradually improves to about 70 by the end of the iterations. This analysis indicates that LLAMA V2 produces more explainable policies compared to the baseline model, with a noticeable gap in scores that widens over time. The higher and more consistent rise in explainability suggests that LLAMA V2 not only generates better decision policies but also provides more interpretable and understandable explanations for these decisions. This is particularly valuable in applications requiring transparency and interpretability in decision-making processes.

**Figure 9:**
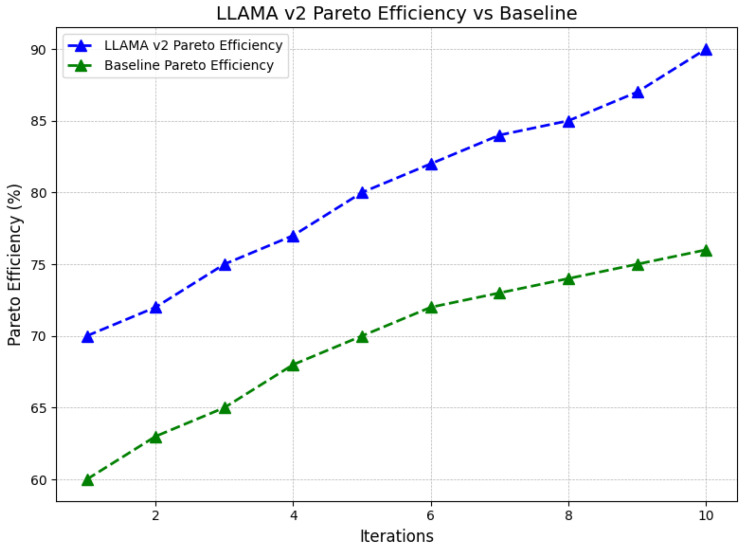
Pareto efficiency of LLAMA V2 compared to baseline over iterations.

The graph in Figure 9 illustrates the Pareto efficiency of LLAMA V2 against a baseline in terms of loss for 10 trials. The x-axis is labeled iterations, while the y-axis represents Pareto efficiency in percentages. As seen in the experiment, in each iteration, the Pareto efficiency of LLAMA V2 is higher than that of the baseline model. LLAMA V2 starts with a recall of about 70 percent and gradually rises to about 90 percent at the tenth iteration. The baseline model starts at about sixty percent and grows more gradually, reaching only just over seventy-five percentiles by the tenth iteration. The evaluation shows that, the further the iterations move from the baseline, the more significant an improvement in values LLAMA V2 can provide. The overall trend of this divergence implies that within LLAMA V2, resource allocation is optimized to a more significant degree, resulting in performance enhancement, while iterations grow, the advantages of relying on LLAMA V2 in finding the best solution that corresponds to various objectives and avoids necessary trade-offs are also accentuated, proving the model’s superiority.

**Figure 10:**
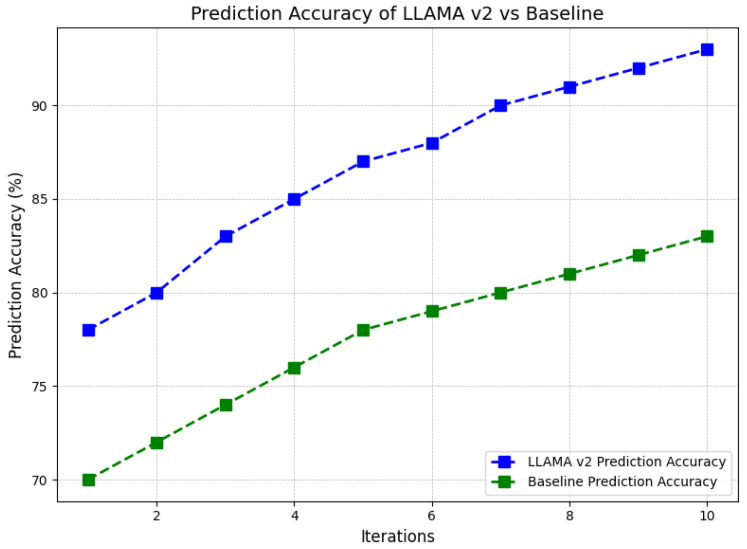
Comparison of the prediction accuracy of LLAMA V2 against a baseline model over multiple iterations for improved performance assessment.

The number of iterations across the graph and the prediction accuracy of the LLAMA V2 against the baseline model are presented below. In the cold-start scenario, LLAMA V2 performs better than the baseline in each iteration, as seen from the initial iterations. The performance of LLAMA V2 rises from 80% in the first stage and reaches 90% in the tenth stage, whereas the baseline increases from nearly 70% to almost 80%. Over the iterations, the architecture of the LLAMA V2 is illustrated in Figure 10 to overtake the baseline, and the difference between the two models increases as the iteration increases. The faster learning curve in accuracy for LLAMA V2 indicates that it is better at adapting to changes and providing better prediction over time.

**Figure 11:**
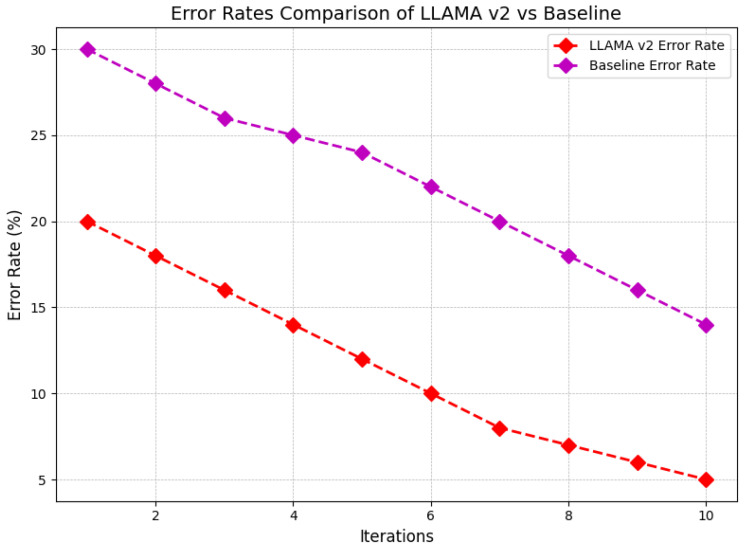
Comprehensive comparison of the error rates between LLAMA V2 and a baseline model across multiple iterations to assess performance improvements and model reliability.

Figure 11 compares the error rates of LLAMA V2 and the baseline model over 10 iterations, showing a consistent decrease in error rates for both. The average accuracy of the LLAMA V2 is initiated at 20%, progressively declining to 5% at the tenth instance; on the other hand, the average baseline model is initiated at 30% and reduces to 15%. In the case of the iterations, the model is seen to again have a lower error rate throughout the iterations, with the difference increasing with each iteration. This implies that LLAMA V2 is better placed to adapt more than the baseline, whereby the error improvement rate is faster.

The analysis shows that LLAMA V2 consistently outperforms the baseline in several vital areas, such as accuracy, explainability, Pareto efficiency, and error rates. The model adapts quickly and improves significantly with each iteration, demonstrating better efficiency and flexibility. This study evaluates essential metrics for decision making and resource allocation in O-RAN systems, emphasizing critical aspects of modern 5G applications, including real-time processing, reliability, and transparency. Incorporating advanced optimization techniques and leveraging LLAMA V2’s strengths demonstrate that LLAMA V2 excels in tasks requiring high accuracy, reliability, and explainability. As such, the study offers valuable insights into addressing the challenges of dynamic and efficient resource allocation in O-RAN systems, making it a significant contribution to wireless communications.

## 7. Conclusions

The study effectively demonstrates the capability of LLAMA V2 to optimize decision-making and resource allocation within O-RAN systems, particularly for network slicing. LLAMA V2 consistently outperforms baseline LLM, AI, and ML models in terms of decision accuracy, explainability, Pareto efficiency, and error reduction across iterations. A formal analysis of the results highlights LLAMA V2’s adaptability, customizability, and efficiency for real-time network management in multi-vendor environments. However, future challenges, such as scalability and the practicality of deployment in diverse operational contexts, must be addressed to ensure sustained performance. The proposed framework offers a comprehensive solution for dynamic multi-vendor challenges faced by wireless networks across 5G and beyond, where AI-driven intelligence plays a foundational role in maintaining system performance and adaptability.

## Figures and Tables

**Table 1:** Comparison of LLAMA V2 with other AI models for O-RAN systems.

Model/Feature	Superior Optimization	Explainable Policies	Real-Time Slicing
LLAMA V2	✓	✓	✓
GPT-4	✓	✓	×
GPT-NeoX	✓	×	×
DQN	×	×	✓
PPO	✓	✓	×
FRL	×	×	×
Actor-Critic	×	×	✓
TL-DRL	✓	✓	×

**Table 2:** Comparison of state-of-the-art LLMs for O-RAN system and resource allocation.

Model	Memory Efficiency	Reasoning Capability	Suitability for O-RAN
LLAMA V2 *	High	High	Best for resource allocation, low latency
GPT-4	Low	Superior	Too resource-intensive for real-time systems
GPT-NeoX	Moderate	Good	Decent, but high memory demand
OPT	Moderate	Moderate	High resource use, slow for real-time
BLOOM	Low	Moderate	High resource usage, slower real-time performance
PaLM	Low	High	Too large, slow for real-time O-RAN
T5	High	Good	Lacks resource efficiency for O-RAN
Grok	High	Good	Good for specific tasks, less flexible
GLaM	Low	High	Infeasible for real-time O-RAN tasks
Jurassic-1	Low	High	Resource-intensive, slow adaptation
BERT	High	Moderate	Good for text understanding, lacks dynamic reasoning
RoBERTa	High	Moderate	Similar to BERT, lacks adaptability
DistilBERT	Very High	Lower than BERT	Too simplistic for complex O-RAN tasks
GPT-2	Moderate	Moderate	Not scalable for large O-RAN resource allocation
CodeGen	Moderate	High for coding tasks	Not optimized for O-RAN systems
XGLM	High	Moderate	Less efficient for multilingual O-RAN
MT-NLG	Low	Very High	Too large for real-time systems
Megatron-Turing	Low	High	Excellent but impractical for real-time O-RAN
ERNIE 3.0	High	Good	Decent for complex reasoning, slower than LLAMA V2
Switch-C	Low	High	Too large, unsuitable for O-RAN applications

* LLAMA V2 is for its superior suitability for O-RAN tasks.

**Table 3:** Comparison of network slicing techniques for O-RAN systems.

Technique	Memory Efficiency	Reasoning Capability	Suitability for O-RAN
Proposed Approach *	High	High	Best for real-time slicing, fast adaptation, low latency
Deep Q-Network (DQN)	Moderate	Moderate	Moderate, slower convergence in dynamic environments
Proximal Policy Optimization (PPO)	Low	High	Suitable for real-time, but requires careful tuning
Federated Learning (FRL)	Moderate	Low	Limited due to slow convergence
Actor-Critic (AC)	Moderate	High	Prone to instability in dynamic slicing
Transfer Learning (TL-DRL)	High	High	Effective for complex O-RAN slices, but needs pre-training
Tabular RL	Low	Low	Not efficient for large O-RAN scenarios
Evolutionary DRL	Moderate	Moderate	Suitable for training, but not for real-time slices
Crowding Game Approach	High	High	Limited by computational overhead
Safe RL	Moderate	Moderate	Good for certain cases, but limited exploration risks
Hybrid Transfer DRL	High	High	Better for practical live deployment but complex
Multi-Agent RL	Moderate	Moderate	Good for collaboration, but slower convergence
GAN-based DRL	High	Moderate	Suitable but prone to instability and slower learning
Latency-aware DRL	Low	High	Excellent for URLLC slices, but lacks generalization
Channel-Aware Scheduling	Moderate	Low	Limited adaptability, good for static environments

* LLAMA V2-based DRL is used for its superior suitability for O-RAN tasks like real-time slicing, fast adaptation, and low latency; these are not fully addressed by other methods.

**Table 4:** Symbols and definitions used in mathematical framework.

Symbol	Description
$X_{i}^{k}\left(t,x\right)$	State variable of network element *i* at time *t* and location *x* in slice *k*.
$\mu_{i}^{k}$	Drift term representing the deterministic evolution of the network state.
$\sigma_{ij}^{k}$	Volatility term capturing stochastic fluctuations in the network state.
$W_{j}\left(x\right)$	Wiener process modeling random noise in the system.
$\nu_{ij}^{k}$	Perturbation term accounting for random disturbances in the network.
L(θ)	Loss function used to train the LLAMA_V2 model.
$E_{u^{k}}$	Expectation operator over control inputs $u^{k}$.
$u_{kk}$	Control policy parameters for resource allocation in slice *k*.
${\mathrm{Cost}}_{i}^{k}\left(u\right)$	Cost function associated with control decisions in slice *k*.
λ,α	Regularization parameters for balancing loss components.
$L^{k}$	Differential operator representing interactions within slice *k*.
$\nabla_{\theta }$	Gradient operator with respect to model parameters θ.

## Data Availability

Data is contained within the article.

## List of Abbreviations

Abbreviation	Meaning
O-RAN	open radio access network
LLAMA V2	Large Language Model Architecture V2
eMBB	enhanced mobile broadband
URLLC	ultra-reliable low-latency communication
mMTC	massive machine-type communication
AI/ML	artificial intelligence/machine learning
RIC	RAN Intelligent Controller
QoS	quality of service
SPDE	stochastic partial differential equation
HJB	Hamilton–Jacobi–Bellman equation
LSTM	long short-term memory
KKT	Karush–Kuhn–Tucker conditions
DRL	deep reinforcement learning
PDE	partial differential equation
NS-3	Network Simulator 3
GPU	graphics processing unit
CP	CANDECOMP/PARAFAC tensor decomposition
IoT	Internet of Things
